# Therapeutic Effect of Ginsenoside Rd on Experimental Autoimmune Encephalomyelitis Model Mice: Regulation of Inflammation and Treg/Th17 Cell Balance

**DOI:** 10.1155/2020/8827527

**Published:** 2020-12-17

**Authors:** Bo Jin, Chixiao Zhang, Yu Geng, Mei Liu

**Affiliations:** ^1^Department of Neurology, Zhejiang Provincial People's Hospital, People's Hospital of Hangzhou Medical College, Hangzhou 310014, China; ^2^Key Laboratory of Neuropsychiatric Drug Research of Zhejiang Province, Hangzhou Medical College, Hangzhou 310013, China

## Abstract

Multiple sclerosis (MS) is an autoimmune inflammatory disease. Inflammatory infiltrates and demyelination of the CNS are the major characteristics of MS and its related animal model-experimental autoimmune encephalomyelitis (EAE). Immoderate autoimmune responses of Th17 cells and dysfunction of Treg cells critically contribute to the pathogenesis of MS and EAE. Our previous study showed that Ginsenoside Rd effectively ameliorated the clinical severity in EAE mice, but the mechanism remains unclear. In this study, we investigated the therapeutic effect of Ginsenoside Rd on EAE *in vivo* and *in vitro* and also explored the potential mechanisms for alleviating the injury of EAE. The results indicated that Ginsenoside Rd was effective for the treatment of EAE in mice and splenocytes. Ginsenoside Rd treatment on EAE mice ameliorated the severity of EAE and attenuated the characteristic signs of disease. Ginsenoside Rd displayed the therapeutic function to EAE by modulating inflammation and autoimmunity, via the downregulation of related proinflammatory cytokines IL-6 and IL-17, upregulation of inhibitory cytokines TGF-*β* and IL-10, and modulation of Treg/Th17 imbalance. And the Foxp3/ROR*γ*t/JAK2/STAT3 signaling was found to be associated with this protective function. In addition, analysis of gut microbiota showed that Ginsenoside Rd also had modulation potential on gut microbiota in EAE mice. Based on this study, we hypothesize that Ginsenoside Rd could be a potential and promising agent for the treatment of MS.

## 1. Introduction

Multiple sclerosis (MS) is an autoimmune inflammatory disease affecting the central nervous system (CNS) of the body [1]. The clinical features of MS are diverse and mainly include limb weakness, paresthesia, fatigue, blurred vision, ataxia, and cognitive deficits [2]. The etiology of MS is thought to result from the interaction of genetic and environment factors, but this remains to be partially understood. CD4^+^ T cells regulated autoimmune/inflammatory response is thought to critically associate with this disease. T cells enter into the CNS through the damaged blood-brain barrier (BBR), initiate a series chain-type inflammatory response, to induce inflammation, demyelization, oligodendrocyte loss, and subsequent axonal and neuronal damage [3]. Treatment options of MS have become increasingly multifaceted and multiselective, but in long-term prognosis, the relapses of MS still cause substantial disability in many patients; a meta-analysis also shows that suicide is significantly associated with MS [4, 5]. Thus, therapeutic strategy that reducing the occurrence and severity of MS to improve prognosis is worthy to be explored. For the study of MS, rat experimental autoimmune encephalomyelitis (EAE) is the most widely used animal model. While it is not a so perfect set of animal model, it can mimic mainly pathological features of MS and can be used for pathophysiology and therapy study [6].

T helper 1 (Th1), T helper 2 (Th2), T helper 17 (Th17) cells, and regulatory T (Treg) cells are critical subsets of CD4^+^ T cells which are central elements in immune homeostasis. Many previous studies show that the imbalance of T cell responses, such as proinflammatory Th1 and Th17 cells, and anti-inflammatory Th2 and Treg cells, is crucial for the pathogenesis and progression of MS and EAE [7–10]. The immoderate immunoreaction of Th17 cells and dysfunction of Treg cells is responsible to dysregulated immunity, inflammatory response, oxidative stress, attack on self-myelin basic protein (MBP) of the MS. Thereby, upregulation of anti-inflammatory Th2 and Treg cells and inhibition of proinflammatory Th1 and Th17 cells, to restore the balance of T cell response, are an ideal strategy for EAE therapy. In addition, for Th17 cells, ROR-*γ*t is the essential transcription factor of it. ROR-*γ*t could be activated by JAK2/STAT3 signaling and thereby promotes the CD4+ T cells differentiating into Th17 cells [11, 12]. Foxp3 is a key regulator of Treg cells; stable expression of Foxp3 is important for the cell development and function of Treg cells [10]. Fox3 can inhibit the differentiation of Th17 cells through the direct interaction with ROR-*γ*t. Other study also shows that modulation of JAK2/STAT3 signaling activity could regulate the balance of Th17 and Treg cells [13, 14].

Ginsenoside Rd is a dammarane-type steroid glycoside extracted from a traditional Chinese herbal medicine, Pana notoginseng (*Panaxnotoginseng* (*Burk.*) *F.H.Chen*). Accumulation studies evidence the multiple pharmacological effects of Ginsenoside Rd, including the application in inflammatory diseases, metabolic diseases, immune regulation, and CNS [15–17]. Particularly, Ginsenoside Rd is a promising neuroprotective agent. It can attenuate the cerebral ischemia-induced BBB damage and spinal cord injury-induced secondary injury through reversing the redox-state imbalance, inhibiting the inflammatory response and apoptosis in the spinal cord tissue of rat model [18, 19]. Other study shows that Ginsenoside Rd has the ability to regulate the Th1/Th2 immune responses [20]. Our previous study of Ginsenoside Rd on EAE animal model shows that Ginsenoside Rd effectively ameliorated the clinical severity in EAE mice, reduced the permeability of the BBB, regulated the secretion of IFN-*γ* and IL-4, and promoted the Th2 shift in cerebral cortex and splenocytes [21]. It indicated the potential of Ginsenoside Rd in inhibiting the clinical course of EAE, but the underlying mechanism remains unclear. Therefore, in this study, we further investigate the therapeutic effect of Ginsenoside Rd in EAE *in vivo* and *in vitro* and also hope to clarify the potential mechanisms underlying the efficacy of Ginsenoside Rd in alleviating the injury of EAE. Based on this study, we hypothesize that Ginsenoside Rd could be a potential and promising agent for MS treatment.

## 2. Materials and Methods

### 2.1. Animals and Regents

C57BL/6 mice, 6 ~ 8 weeks of age, were purchased from the Shanghai SLAC Laboratory Animal Co. Ltd. (Certificate No. SCXK (Hu) 2013-0018; Shanghai, China) and housed under specific pathogen-free conditions with a 12 h light/dark cycle. The mice have free access to food and water, and all experimental procedures were conducted in accordance with the Institutional Animal Care and Use Committee Guidelines of Zhejiang Chinese Medical University (Hangzhou, China).

Ginsenoside Rd with a purity of 98% was purchased from Nanjing Zelang Medical Technology (Nanjing, China). Myelin oligodendrocyte glycoprotein (MOG)_35-55_ peptide was synthesized by Meilian Biotechnological Co., Ltd. (Xi'an, China), and the purity of the peptide was greater than 95%. *Mycobacterium tuberculosis* H37RA was obtained from Difco (Germany). Pertussis toxin (PTX) was purchased from Alexis (San Diego, CA). Complete Freund's adjuvant (CFA) was purchased from Sigma-Aldrich (St. Louis, USA).

### 2.2. EAE Induction and Treatment

The induction of EAE was performed by C57BL/6 mice immunized with a MOG peptide, and the protocols were referenced to the published study [20]. Briefly, 200 *μ*g Mog_35-55_ peptide was emulsified in CFA containing 500 *μ*g *Mycobacterium tuberculosis* H37RA and percutaneous injected to C57BL/6 mice. This protocol was repeat at 7 days later. At the same time, mice were also i.p. injected with 0.1 ml PBS containing 300 ng PTX, and this protocol was repeat after 48 h. At day 13 of immunization, Ginsenoside Rd was dissolved into PBS and i.p. injected to the EAE mice at a dosage of 40 mg/kg/d. The dosage of Ginsenoside Rd was referenced to our previous study [21]. Mice in control group (*n* = 10) were percutaneous and injected with same volume of PBS (i.p.). During the day of 0 to 35 of the experiment, mice in EAE group (*n* = 10) and EAE + Ginsenoside Rd group (*n* = 10) were examined daily for disease signs by clinical scores. Researchers were blinded to experimental conditions and assigned scores on a scale of 0 ~ 5 as follows [22]: 0, no signs; 1, loss of tail tonicity; 2, flaccid tail; 3, ataxia and/or paresis of hind limbs; 4, complete paralysis of hind limbs; and 5, moribund or death.

### 2.3. Tissue Preparation and Splenocyte Isolation

On the day 20 postimmunization, 6 mice in each group were euthanized; the blood was collected from the abdominal cavity and centrifugated at 2,500 rpm for 15 min to obtain the serum sample. The lumbar spinal cords were harvested after PBS perfusion and immediately frozen in liquid nitrogen and stored at -80°C. Splenic cells were also isolated from the spleen tissues of different groups on day 20 postimmunization. Briefly, spleen tissues in each group were removed and ground; then, the chopped spleen tissues were passed through a 70 *μ*m nylon mesh cell strainer, washed with RPMI-1640, to obtain a splenocytes suspension. Then, the cells (2 × 10^6^/well) in each group were incubated in RPMI 1640 medium containing 10% FBS. 10 *μ*g/ml of MOG_35-55_ was added for immunization, and cell supernatants were collected after 48 h for further ELISA and flow cytometry assay.

### 2.4. Histopathological Assessment

The spinal cord tissues were fixed in 4% paraformaldehyde and embedded in paraffin to cut into 5 *μ*m sections. Then, the spinal cord sections were stained with hematoxylin and eosin (H&E) for routine evaluation of histopathological changes and inflammation and were stained with luxol fast blue (LFB) for the evaluation of demyelination. In addition, semiquantitative analysis was also used to assess the degree of inflammation and demyelination. A 0-3 scale was used to assess the HE stained sections for inflammation [23]: 0, no inflammatory cells; 1, a few scattered inflammatory cells; 2, organisation of perivascular inflammatory infiltrates; and 3, extensive perivascular cuffing with extension into adjacent parenchyma or parenchymal infiltration without obvious cuffing. Demyelination in the LFB stained spinal cords was scored according to the following [24]: 1, traces of subpial demyelination; 2, marked subpial and perivascular demyelination; 3, confluent perivascular or subpial demyelination; 4,massive perivascular and subpial demyelination involving one half of the spinal cord with presence of cellular infiltrates in the CNS parenchyma; and 5, extensive perivascular and subpial demyelination involving the whole cord section with presence of cellular infiltrates in the CNS parenchyma.

### 2.5. Immunofluorescence Staining

The expression of MBP in spinal cord tissues was also evaluated using immunofluorescence staining. The spinal cord sections in each group were firstly incubated in blocking solution (3% donkey serum, 0.3% Triton X-100 in PBS) for 30 min and stained with primary antibodies anti-MBP (dilution ratio, 1 : 200; Abcam, UK) at 4°C overnight. Then, the sections were further incubated with secondary antibody to goat anti-rabbit. DAPI was used to stain the DNA in the nucleus. Sections were imaged by a Nikon Eclipse Ti-SR inverted fluorescence microscopy (Nikon, Japan), and the quantification of the fluorescence intensity was analyzed by the ImageJ software.

### 2.6. Quantitative Real-Time PCR Analysis (qRT-PCR)

Total DNA was isolated from the collected feces using QIAamp kit (QIAGEN, Germany) according to the manufacturer's instructions. qRT-PCR was performed using the Applied Biosystems 7500 Real-Time PCR System with LightCycler® 96 Real-time PCR (Roche, Switzerland). The reaction condition was 95°C, 15 s, 60°C, 60 s, and 40 circles. The sequences of the primers are listed in Table 1.

### 2.7. Treatment in Splenocytes

The isolated splenocytes from the EAE group were cultured in RPMI-1640 medium supplemented with 10% FBS in a 5% CO_2_ incubator at 37°C. Then, the splenocytes were divided into two groups: vehicle group and Ginsenoside Rd group. The vehicle group splenic cells were incubated with 10 *μ*g/ml of MOG_33-35_ for 48 h. The Ginsenoside Rd group splenic cells were incubated with 10 *μ*g/ml of MOG_33-35_ and Ginsenoside Rd (50 *μ*M) for 48 h.

### 2.8. ELISA Assay

The concentrations of IL-17, IL-6, TGF-*β*, and IL-10 in the serum samples and the supernatants of splenocytes were determined using ELISA assay with the corresponding cytokine-specific detection kits, following the manufacturer's instructions.

### 2.9. Flow Cytometry Assay

Splenocytes were used to analyze the CD4^+^ T cells. First, splenocytes were incubated with fluorochrome-conjugated antibodies (Abs) to CD4, CD25 (BioLegend, USA), or isotype control Abs for 30 min on ice to make surface-marker stain. Splenocytes were then stimulated with 50 ng/ml phorbol myristate acetate (PMA) and 500 ng/l ionomycin in the presence of Golgi-Plug for 5 h. After cell surface-stained, fixed, and permeabilized, intracellular cytokines staining was conducted, and cells were stained with Abs to IL-17A (BioLegend, USA) for Th17 cells and Foxp3 (BioLegend, USA) for Treg cells, respectively. Then, followed by flow cytometry analysis using a flow cytometer (C6, BD, USA) and data were analyzed with the FlowJo software Vision 10 (Beckman).

### 2.10. Western Blotting Analysis

To investigate the expressions of ROR-*γ*t/Fox3/JAK2/STAT3 signaling in the lumbar spinal cord and splenocytes of differently treated groups, western blot analysis was performed. The spinal cord tissues or splenic cells were lysed with RIPA lysis buffer (Beyotime, China) in ice-cold, and the concentrations of proteins in tissues or cells were measured using a BCA protein assay reagent kit (Solarbio, China). Then, equal amounts of protein samples were loaded on 10% gradient sodium dodecyl sulfate-polyacrylamide gels (SDS-PAGE) for electrophoresing and transferred onto polyvinylidene fluoride (PVDF) membrane. The membranes were blocked with 5% nonfat milk, washed with TBST buffer three times, and then followed by incubation overnight with primary antibodies (anti-JAK1 antibody: 1 : 5000, anti-JAK2 antibody: 1 : 5000, anti-FOXP3 antibody: 1 : 1000, anti-ROR*γ*t antibody: 1 : 2000, anti-STAT3 antibody: 1 : 1000, anti-GAPDH antibody: 1 : 5000; Abcam, UK; anti-phospho-JAK1 (Tyr1125) antibody: 1 : 1000, anti-phospho-JAK2 (Tyr931) antibody: 1 : 1000, anti-phospho-STAT3 (Tyr705) antibody: 1 : 1000; Affinity, USA) at 4°C. After the incubation and washed with TBST buffer three times, the membranes were incubated with goat anti-rabbit-HRP for 30 min. Then, the enhanced chemiluminescence reagents were employed to detect chemiluminescence signals; the protein bands were quantified using the ImageJ software. The experiment was repeated in triplicate and used GAPDH as internal control.

### 2.11. Statistical Analysis

Data are expressed as the means ± standard deviation (SD). Differences between the two groups were analyzed using Student *t*-test; differences among the more than two groups were analyzed by conducting one-way ANOVA followed by analysis followed by SNK test using the SPSS 18.0 software (SPSS, USA). *P* values < 0.05 were considered statistically significant.

## 3. Results

### 3.1. Treatment with Ginsenoside Rd Ameliorated the Clinical Severity of EAE Mice

**Figure 1:**
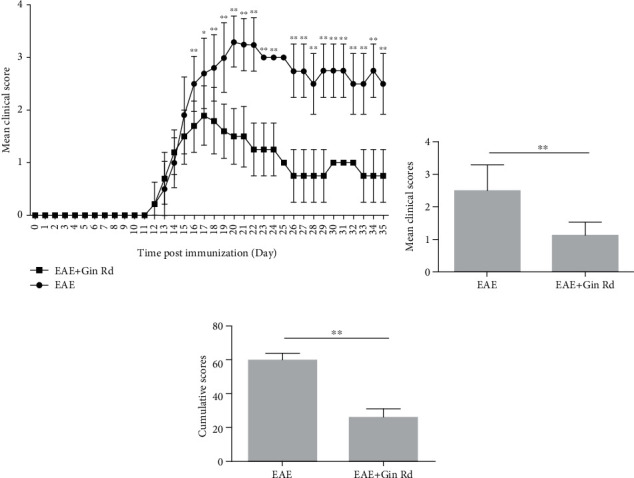
Treatment with Ginsenoside Rd ameliorated the clinical severity of experimental autoimmune encephalomyelitis (EAE) mice. Following immunization and treatment with Ginsenoside Rd, daily clinical scores of EAE mice with or without Ginsenoside Rd treatment were recorded over the course of the 35-day experiment: (a) daily clinical scores of EAE mice with or without Ginsenoside Rd treatment; (b) mean clinical scores of EAE mice with or without Ginsenoside Rd treatment; (c) cumulative clinical scores of EAE mice with or without Ginsenoside Rd treatment. ^∗^*P* < 0.05, ^∗∗^*P* < 0.01 vs. EAE group. Gin Rd: Ginsenoside Rd.

First, the potential effect of Ginsenoside Rd on clinical course of EAE was assessed. Following immunization and with Ginsenoside Rd treatment or vehicle alone, daily clinical scores of EAE mice were recorded over the course of the 35-day experiment. As showed in Figure 1(a), the EAE model group and EAE + Gin Rd group of mice began to develop clinical signs of EAE about on day 11 postimmunization, and the scores were increased continuously for several days, maintained for several days, and then decreased. As a result, the daily clinical scores in the EAE + Gin Rd group were lower than those in the EAE model group mice. Moreover, the mean clinical score and cumulative score in the EAE + Gin Rd group were all significantly lower than those in the EAE model group of mice (Figure**s**1(b) and 1(c)).

### 3.2. Treatment with Ginsenoside Rd Improved the Histopathology Outcomes of EAE Mice

**Figure 2:**
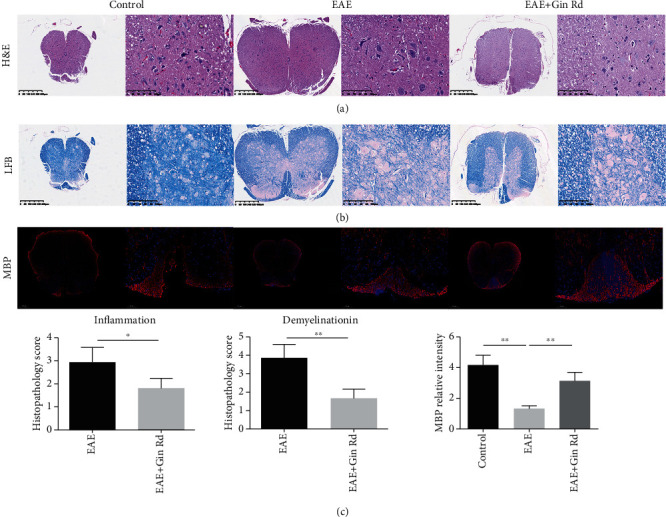
Ginsenoside Rd treatment ameliorated the severity of EAE via inhibiting spinal cord inflammation and demyelination. Following immunization and treatment with Ginsenoside Rd, some mice from the control, EAE, and Ginsenoside Rd-treated EAE groups were subjected to histopathological assay at day 20 postimmunization. Spinal cord tissue sections were stained with H&E or LBP, respectively. In addition, the degrees of inflammatory infiltrates and demyelination were semiquantitatively analyzed. Data are expressed as the mean ± SD for each group (*n* = 3). (a) H&E analysis of the spinal cord sections. Magnification ×40, 200. (b) LBP analysis of the spinal cord sections. Magnification ×40, 200. (c) MBP immunofluorescence staining of the spinal cord sections. Tissue sections of spinal cord from each group mice were stained with antibodies against MBP (red) and DAPI (blue, nucleus). Magnification ×50, 200. ^∗^*P* < 0.05, ^∗∗^*P* < 0.01 vs. EAE group. Gin Rd: Ginsenoside Rd.

The histopathology changes of spinal cord from different groups of mice were also examined on day 20 postimmunization. H&E and LBP staining were performed to detect the spinal cord inflammatory infiltrates and demyelinationin of mice, respectively. H&E staining (Figure 2(a)) showed that there were numerous inflammatory cells infiltrated in the spinal cord tissue sections of the EAE group compared to the normal control group; semiquantitative analysis also showed that the inflammation degree was significantly increased. While in the Ginsenoside Rd-treated group, the infiltration of inflammatory cells was alleviated; the histopathology score of inflammation was also significantly lower compared to the EAE group. LBP staining (Figure 2(b)) showed that there was demyelinationin in the spinal cord tissues of EAE mice; semiquantitative analysis revealed that the demyelinationin score was significantly increased compared to the control mice. With the treatment of Ginsenoside Rd, the degree of demyelinationin was alleviated; the pathological scores of demyelination were also lower than that in the EAE mice. MBP staining also showed that the relative expression of MBP was decreased in the EAE group of mice, and Ginsenoside Rd treatment increased the MBP expression and blocked myelin loss compared to the EAE group.

### 3.3. Ginsenoside Rd Treatment Suppressed the Production of Inflammatory Cytokines in EAE Mice

**Figure 3:**
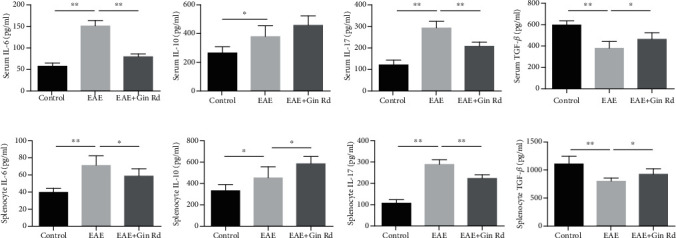
The concentrations of IL-6, IL-10, IL-17, and TGF-*β* in serum and splenocyte supernatants of mice (*n* = 6). Following immunization and treatment with Ginsenoside Rd, some mice from the control, EAE, and Ginsenoside Rd-treated EAE groups were sacrificed at day 20 postimmunization. Serum was collected, and splenocytes were isolated, for ELISA assay to detect the concentrations of IL-6, IL-10, IL-17, and TGF-*β*. Data are expressed as mean ± SD for each group (*n* = 3). (a) ELISA analysis for the levels of IL-6, IL-10, IL-17, and TGF-*β* in serum of mice. (b) ELISA analysis for the levels of IL-6, IL-10, IL-17, and TGF-*β* in splenocyte supernatants of mice. ^∗^*P* < 0.05, ^∗∗^*P* < 0.01 vs. EAE group. Gin Rd: Ginsenoside Rd.

The concentrations of IL-6, IL-10, IL-17, and TGF-*β* in serum and splenocyte supernatants of mice were detected with ELISA assay, and the results were showed in Figure 3. As the results indicated, compared to the control group, the concentrations of serum IL-6, IL-10, and IL-17 were increased significantly; TGF-*β* was decreased significantly (Figure 3(a)). With the treatment of Ginsenoside Rd and compared to the EAE group, it could be found that the concentrations of serum IL-6 and IL-17 were lowered significantly, but TGF-*β* was increased significantly. For the inflammatory cytokine levels in the splenocyte supernatants of mice, as shown in Figure 3(b), compared to the control group, the IL-6, IL-10, and IL-17 levels were increased significantly, but TGF-*β* was decreased significantly, while Ginsenoside Rd treatment significantly lowered the levels of IL-6 and IL-17 and increased the levels of IL-10 and TGF-*β* significantly compared to those in the EAE group.

### 3.4. Effect of Ginsenoside Rd Treatment on Treg/Th17 Cell Imbalance in EAE Mice

**Figure 4:**
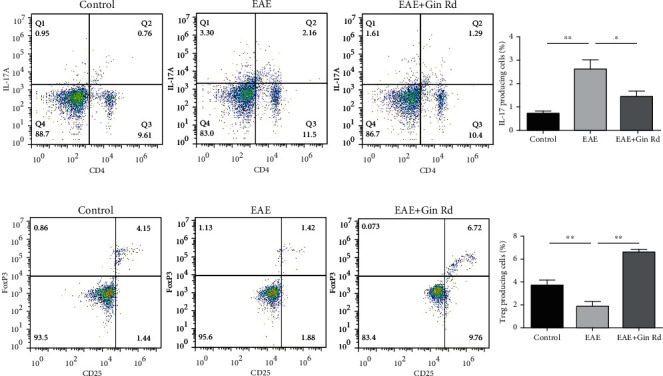
Ginsenoside Rd treatment modulates Treg/Th17 cell imbalance in EAE mice. Following immunization and treatment with Ginsenoside Rd, some mice from the control, EAE, and Ginsenoside Rd-treated EAE groups were sacrificed at day 20 postimmunization; splenocytes were isolated. The percentages of CD4+ IL-17+ Th17 cell (a) and CD4+ CD25+ Foxp3+ Treg cells (b) in each group were determined by flow cytometry (*n* = 3). Data are expressed as the mean ± SD for each group (*n* = 3). ^∗^*P* < 0.05, ^∗∗^*P* < 0.01 vs. EAE group. Gin Rd: Ginsenoside Rd.

Further, we examined the effect of Ginsenoside Rd on T cell immunity and inflammation in EAE mice. Flow cytometry analysis revealed that the percentages of CD4+ IL-17+ Th17 cells in splenocytes of EAE mice were significantly increased than that in normal controls, but in the Ginsenoside Rd treatment group, the Th17 cell percentage was significantly lowered (Figure 4(a)). For Treg cells, flow cytometry analysis showed that in splenocytes of EAE mice, the percentages of CD4+ CD25+ Foxp3+ Treg cells were significantly increased, but in the Ginsenoside Rd treatment group, the Treg cell percentage was increased significantly (Figure 4(b)).

### 3.5. Gut Microbiota Changes in EAE Mice

**Figure 5:**
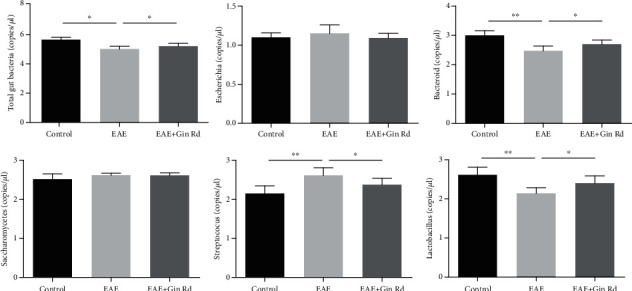
Relative content of gut bacteria in different groups by qRT-PCR analysis. Data are expressed as the mean ± SEM of each group (*n* = 3). ^∗^*P* < 0.05, ^∗∗^*P* < 0.01 vs. EAE group. Gin Rd: Ginsenoside Rd.

The changes of gut microbiota in EAE mice were also detected. As showed in Figure 5, compared to the control mice, the relative contents of total gut microbiota, *bacteroid*, and *lactobacillus* were significantly decreased; *streptococcus* was significantly increased in the EAE group of mice. In the EAE + Gin Rd group treated with Ginsenoside Rd, the relative contents of total gut microbiota, *bacteroid*, and *lactobacillus* were significantly increased; *streptococcus* was decreased compared to those in the EAE group.

### 3.6. The Effect of Ginsenoside Rd Treatment on the Foxp3/ROR*γ*t/JAK2/STAT3 Signaling Pathway in EAE Mice

**Figure 6:**
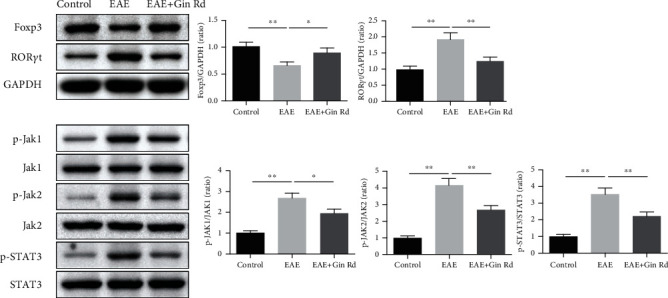
Relative protein expressions of Foxp3, ROR*γ*t, p-JAK1, JAK1, p-JAK2, JAK2, p-STAT3, and STAT3 in the spinal cord of EAE mice using western blotting assay (*n* = 3). GAPDH was used as the internal control. Data are expressed as the mean ± SD for each group (*n* = 3). ^∗^*P* < 0.05, ^∗∗^*P* < 0.01 vs. EAE group. Gin Rd: Ginsenoside Rd.

As the above results suggested that Ginsenoside Rd could regulate inflammation and immune responses in EAE mice, we further investigated the effect of Ginsenoside Rd on the Foxp3/ROR*γ*t/JAK2/STAT3 signaling pathway. Western blot assay was used to detect the protein expression levels of Foxp3, ROR*γ*t, p-JAK1, JAK1, p-JAK2, JAK2, p-STAT3, and STAT3 in the spinal cord of mice (Figure 6). As the results showed, the relative protein expression level of Foxp3 was significantly decreased, while ROR*γ*t was significantly increased in the EAE model group compared to those in the control group. However, Ginsenoside Rd treatment significantly activated the level of Foxp3 and suppressed the level of ROR*γ*t compared to those in the EAE group. Furthermore, the relative expression levels of p-JAK1/JAK1, p-JAK2/JAK2, and p-STAT3/STAT3 in the spinal cord of EAE mice were significantly increased compared to those in the control group, while Ginsenoside Rd treatment inhibited the levels of p-JAK1/JAK1, p-JAK2/JAK2, and p-STAT3/STAT3 significantly.

### 3.7. Treatment with Ginsenoside Rd Inhibited the Inflammation and Improved Treg/Th17 Cell Imbalance in Splenocytes with EAE

**Figure 7:**
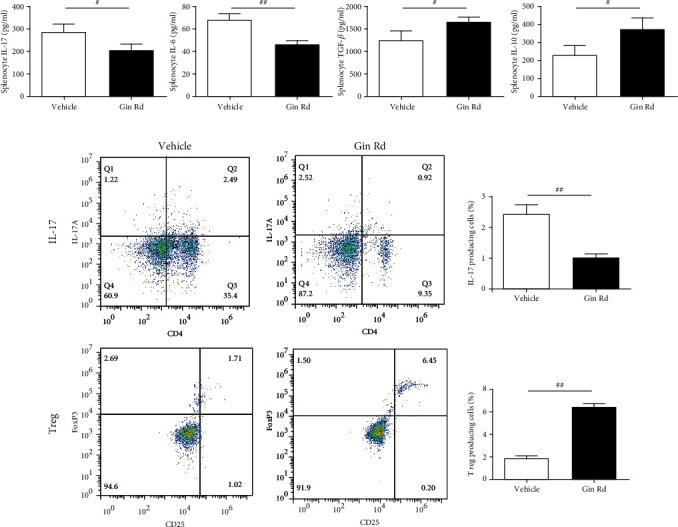
The effect of Ginsenoside Rd on inflammatory cytokine levels and Treg/Th17 cell percentages in splenocytes of EAE. Splenocytes were isolated from EAE mice and further treated with 10 *μ*g/ml of MOG_33-35_ for 48 h, with or without Ginsenoside Rd (50 *μ*M). (a) ELISA assay for detection of the levels of IL-6, IL-10, IL-17, and TGF-*β* in splenocyte supernatants (*n* = 3). (b) Flow cytometry assay for detection the percentages of CD4+ IL-17+ Th17 and CD4+ Foxp3+ Treg cells in splenocytes (*n* = 3). Data are expressed as the mean ± SEM of each group (*n* = 3). ^#^*P* < 0.05, ^##^*P* < 0.01 vs. vehicle group. Gin Rd: Ginsenoside Rd.

ELISA assay results showed that compared to the EAE splenocytes induced with MOG_35-55_, the concentrations of IL-17 and IL-6 were significantly lowered, and the concentrations of TGF-*β* and IL-10 were significantly higher in Ginsenoside Rd-treated and MOG_35-55_-induced EAE splenocytes (Figure 7(a)). Flow cytometry analysis showed that the percentages of CD4+ IL-17+ Th17 cells were significantly lower, and the percentages of CD4+ Foxp3+ Treg cells were significantly higher in Ginsenoside Rd-treated splenocytes than those in vehicle-treated splenocytes of EAE (Figure 7(b)).

### 3.8. Ginsenoside Rd Treatment Regulated the Foxp3/ROR*γ*t/JAK2/STAT3 Signaling Pathway in Splenocytes with EAE

**Figure 8:**
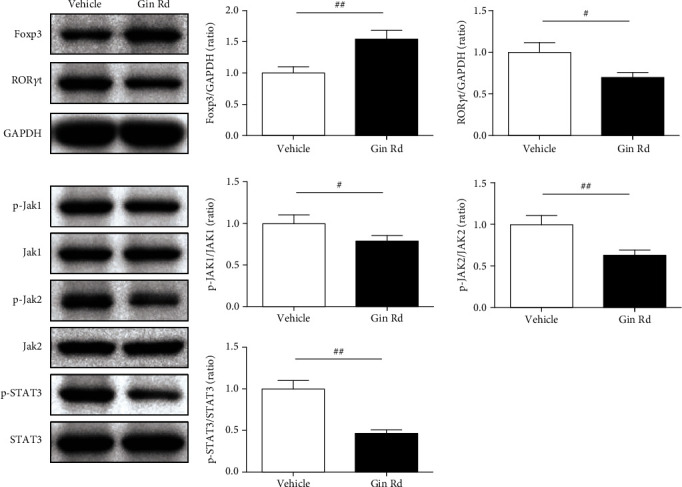
Relative protein expression of Foxp3, ROR*γ*t, p-JAK1, JAK1, p-JAK2, JAK2, p-STAT3, and STAT3 in splenocytes of EAE using western blotting assay (*n* = 3). GAPDH was used as the internal control. Data are expressed as the mean ± SEM of each group (*n* = 3). ^#^*P* < 0.05, ^##^*P* < 0.01 vs. vehicle group. Gin Rd: Ginsenoside Rd.

As the above results suggested that Ginsenoside Rd could also regulate the inflammation and Treg/Th17 cell imbalance in splenocytes with EAE, we further investigated the effect of Ginsenoside Rd on the Foxp3/ROR*γ*t/JAK2/STAT3 signaling pathway. Western blot assay was used to detect the protein expression levels of Foxp3, ROR*γ*t, p-JAK1, JAK1, p-JAK2, JAK2, p-STAT3, and STAT3 in splenocytes (Figure 8). As the results showed, the relative protein expression level of Foxp3 was significantly increased, while ROR*γ*t was significantly decreased in Gin Rd group splenocytes compared to those in vehicle group splenocytes. In addition, the relative expression levels of p-JAK1/JAK1, p-JAK2/JAK2, and p-STAT3/STAT3 in splenocytes of the Gin Rd group were significantly suppressed compared to those in the vehicle group without Ginsenoside Rd treatment.

## 4. Discussion

As an autoimmune inflammatory disease, MS typically occurs in young adults and has a high risk of disability in most patients. Treatments for MS are not only costly but also have the risk of side effects. In this study, based on a widely used MS animal model-EAE, we reported that Ginsenoside Rd was effective for the treatment of EAE *in vivo* and *in vitro*. Ginsenoside Rd treatment of EAE ameliorated the severity of EAE and attenuated the characteristic signs of disease. In addition, Ginsenoside Rd displayed the therapeutic function to EAE specifically by modulating inflammation and immunity, via the inhibition of related inflammatory cytokine secretion and regulation of Treg/Th17 cell imbalance. And the Foxp3/ROR*γ*t/JAK2/STAT3 signaling was associated to this function in EAE.

As well acknowledged, inflammatory infiltrates and demyelination of the CNS are the major characteristics of MS and EAE animal model [1, 25]. In this study, histopathological assay by H&E and LBP staining showed that the spinal cord tissues of EAE group mice have significant inflammatory infiltrates and demyelination. And MBP is a major component of myelin sheath in CNS, with the ability of maintaining structure and function of myelin sheath [26, 27]. In particular, MBP is correlated with the severity of MS. MBP immunofluorescence staining intensity in this study was also weakened in spinal cord of EAE mice, which were consisted with the major characteristics of EAE. While in Ginsenoside Rd-treated group mice, the degrees of inflammatory infiltrates and demyelination as well as the MBP expression in spinal cord tissues were restored. These results suggested the therapeutic potential of Ginsenoside Rd to EAE. The clinical scores of EAE mice were also in accordance with these improvements in the Ginsenoside Rd-treated group. As a natural agent, except for the various pharmacological activities, Ginsenoside Rd is also a promising anti-inflammation and neuroprotective agent [15, 28]. Clinical studies in healthy volunteers and in patients with neurological disease or deficit showed that ginsenosides, including Ginsenoside Rd, can affect neurotransmission and neuroprotection [29]. López et al. reported that ginsenosides induce neuroprotection effect on astrocytes mainly through the activation of antioxidant enzymes [30]. In addition, our previous study of Ginsenoside Rd on EAE mice also showed that Ginsenoside Rd effectively ameliorated the clinical severity of EAE mice and reduced the permeability of BBB, which also proved the neuroprotective and therapeutic potential of Ginsenoside Rd on MS [20].

The immoderate autoimmune responses of Th17 cells and dysfunction of Treg cells critically contribute to the pathogenesis of MS and EAE [31]. Th17 cell is a proinflammatory subset of Th cells and contributes to autoimmunity, inflammatory response, and tissue damage; in contrast, Treg cell has anti-inflammatory properties and confers protective effects on CNS [32]. Therefore, modulation of Th17/Treg balance is an ideal strategy for EAE therapy. In this study, except for the amelioration effect of Ginsenoside Rd on clinical severity of EAE, the regulation potentiality on Th17 and Treg cells as well as associated proinflammatory and anti-inflammatory cytokines by Ginsenoside Rd was investigated. As the flow cytometry results indicated, Ginsenoside Rd modulated the percentages of Treg and Th17 cells in splenocytes of EAE and thus improved the Treg/Th17 cell imbalance. Furthermore, Treg and Th17 cell characteristic cytokines, IL-17 and TGF-*β*, as well as IL-6 and IL-10, the characteristic cytokines of Th1/2 cells, their concentrations in serum and splenocytes were also restored with the treatment of Ginsenoside Rd.

The integrity of BBR is an important prerequisite for normal function of CNS, but Th17 cells could disrupt BBR by the action of IL-17. IL-17 can act on endothelial cells directly, sequentially disrupts the BBR tight junctions and integrity, and promotes inflammatory infiltrates and tissue damage of CNS via Th17 lymphocytes recruitment [33, 34]. IL-17 also induces the production of cytokines, such as IFN-*γ*, IL-1, and IL-6 that facilitating inflammatory cell infiltration into the CNS [35]. Thereby, the lower levels of IL-17 production may inhibit the functional development of Th17 cells in EAE. Besides, as a crucial transcriptional regulator of Th17 cell differentiation, the suppression on ROR*γ*t transcription could result in the inhibition on Th17 cell differentiation and function [36]. In present study, we evaluated the differentiation of Th17 cells by examining the percentage of Th17 cells, as well as the production of IL-17 in activated splenocytes of mice with EAE. As a result, Ginsenoside Rd treatment modulated the population of Th17 cells and production of IL-17; the protein expressions of ROR*γ*t in the spinal cord and splenocytes of EAE mice were also decreased, indicated that Ginsenoside Rd inhibited the antigen-specific Th17 responses in EAE via the inhibition of IL-17, IL-6, and ROR*γ*t expression. As a subset of ginsenosides, Park et al. study showed that Ginsenoside Rg3 alleviated the onset and severity of EAE by significantly inhibited the Th17 differentiation and Th17-mediated neuro-inflammation and hampered the expression of IL-17A and ROR*γ*t in T cells [37].

In contrast, Treg cells are negative regulators of autoimmune diseases and play a critical role in the maintenance of self-tolerance. Treg cells induce to the production of inhibitory cytokines TGF-*β* and IL-10, protect against inflammatory infiltrates and tissues damage [38]. A disturbance in the function and percentage of the Treg cells is associated with the severity of disease in relapsing MS [39]. Korean red ginseng extract, which is abundant with ginsenosides, was demonstrated to significantly reduce the population of CD4+, CD4+/IFN-*γ* +, and CD4 + IL-17+ T cells, increase the population of Treg cells in the spinal cord and lymph nodes, corresponding to the downregulation of the mRNA expression of IFN-*γ*, IL-17, and IL-23 and upregulation of the mRNA expression of Foxp3 in the spinal cord of EAE [40]. Kim et al. reported that Ginsenoside Rd could induce Treg cell differentiation by upregulating the Foxp3 expression and increasing the generation of TGF-*β*1, IL-10, and IL-35 [41]. In this study, we found that Ginsenoside Rd treatment modulated the population of Treg cells in activated splenocytes, and productions of TGF-*β*1 and IL-10 were also increased. As a key regulator of Treg cells, the protein expressions of Fox3 in the spinal cord and splenocytes of EAE mice were also activated. Furthermore, the protein expressions of JAK2/STAT2 signaling that is proved to rectify the imbalance of Th17/Treg cells were also regulated with the treatment of Ginsenoside Rd. These results indicated that Ginsenoside Rd upregulated the levels of TGF-*β*1, IL-10, and Fox3; downregulated the levels of IL-17A, IL-6, and ROR*γ*t; and changed the percentage of Th17 and Treg cells in EAE and thus improving Treg/Th17 cell imbalance in EAE.

Furthermore, the present study also investigated the gut microbiota changes in EAE mice. The results showed that the gut microbiota was changed in EAE mice, and Ginsenoside Rd treatment restored partly. The gut microbiota plays an essential role in the occurrence and development of the immune system in EAE; it appears to be involved in modulating the host's immune system, altering the integrity and function of the BBB, triggering autoimmune demyelination, expressing myelin genes, and interacting directly with different cell types in the CNS [42, 43]. Our study showed that the *bacteroid* and *lactobacillus* were decreased in EAE mice, which was consisted with the previous studies [44, 45]. Ginsenoside Rd treatment restored the decreased content of *bacteroid*, *lactobacillus*, and total gut bacteria, indicated a potential regulation effect of on EAE mice. In addition, this indicated that microbiota therapy restoration of the microbial population in patients with MS also provides possibility for MS treatment.

## 5. Conclusions

In summary, based on EAE model induced by MOG_35-55_, this study indicated that Ginsenoside Rd was effective for the treatment of EAE *in vivo* and *in vitro*. Ginsenoside Rd treatment of EAE ameliorated the severity of EAE and attenuated the characteristic signs of disease. In addition, Ginsenoside Rd displayed the therapeutic function to EAE specifically by modulating inflammation and autoimmunity, via the downregulation of related proinflammatory cytokines IL-6 and IL-17, upregulation of inhibitory cytokines TGF-*β* and IL-10, and regulation of Treg/Th17 imbalance. And the Foxp3/ROR*γ*t/JAK2/STAT3 signaling was associated to this protective function of Ginsenoside Rd in EAE. According to this finding, we hypothesized that Ginsenoside Rd could be a potential and promising agent for the treatment of MS.

## Figures and Tables

**Table 1:** Primers for qRT-PCR.

Target bacteria	Forward	Reserve
Total gut bacteria	5′-TCCTACGGGAGGCAGCAGT-3′	5′-GGACTACCAGGGTATCTAATCCTGTT-3′
*Escherichia*	5′-GTTAATACCTTTGCTCATTGA-3′	5′-ACTCGTTCTACTTCCCATTGT-3′
*Bacteroid*	5′-CACGAAGAACTCCGATTG-3′	5′-CACTTAAGCCGACACCT-3′
*Saccharomycetes*	5′-GAAGAGTCGAGTTGTTTGGGAA-3′	5′-TCCTTCCCTTTCAACAATTTCAC-3′
*Streptococcus*	5′-AGCAGTAGGGAATCTTCCA-3′	5′-CGCCACTGGTGTTCYTCCATATA-3′
*Lactobacillus*	5′-AGCAGTAGGGAATCTTCCA-3′	5′-CGCCACTGGTGTTCYTCCATATA-3′

## Data Availability

The data used to support the findings of this study are included within the article.
